# Modulation of Astrocytic Glutamine Synthetase by Endocannabinoid 2-Arachidonoylglycerol in JNK-Independent Pathway

**DOI:** 10.3389/fpain.2021.682051

**Published:** 2021-07-08

**Authors:** Jing Wang, Shenghong Wang, Hua Zhang

**Affiliations:** ^1^School of Clinical Medicine, Southern Medical University, Guangzhou, China; ^2^The People's Hospital of Baoan District, Shenzhen, China; ^3^Department of Orthopaedics, The Affiliated Second Hospital of Lanzhou University, Lanzhou, China

**Keywords:** JNK, cannabinoid receptor, 2-AG, astrocyte, glutamine synthetase

## Abstract

**Background and Objective:** The glutamine synthetase (GS), an astrocyte-specific enzyme, plays an important role in neuroprotection through the glutamate/glutamine shuttle and can be modulated by endocannabinoid (eCB) 2-arachidonoylglycerol (2-AG) through extracellular signal-regulated protein kinase ½ (ERK1/2) and p38 signaling pathways. However, the role of c-Jun N-terminal kinase (JNK) signaling pathway in the modulation of GS in astrocytes by 2-AG is not clear.

**Materials and Methods:** The expression of GS and JNK in astrocytes following the exposure to lipopolysaccharide (LPS) was examined with Western blotting and immunochemistry.

**Results:** The results revealed that short-term exposure to LPS activated GS and increased phosphorylation of JNK in astrocytes in a time-dependent manner. Treatment with 2-AG reversed the changes in GS but had no effect on the activation of JNK.

**Conclusions:** These findings suggest that the activation of JNK induced by LPS is not involved in the modulation of astrocytic GS by 2-AG.

## Introduction

The astrocytic glutamine synthetase (GS) can modulate the extracellular concentration of glutamate by converting glutamate into glutamine and is verified to be involved in a variety of neurological disorders such as neurodegenerative diseases and chronic pain (1). Endocannabinoids (eCBs) are endogenous mediators of lipid signaling with the capabilities to modulate the synaptic function and to provide neuroprotective and anti-inflammatory effects (2). 2-Arachidonoylglycerol (2-AG) is one of the most abundant eCBs and plays a potential role in protecting neurocytes from injuries induced by inflammation and insults of neurodegenerative diseases (3). In addition, previous studies found that 2-AG has the capacity of attenuating neuropathic pain and mechanical hyperalgesia in several preclinical models of chronic pain (4, 5).

The previous study indicates that 2-AG is involved in the modulation of synaptic function, neuroprotection, and stimulation of mitogen-activated protein kinase (MAPK) family by binding to and activating the G-protein-coupled receptors (GPCR), cannabinoid receptor type 1 (CB_1_R), and cannabinoid receptor type 2 (CB_2_R), which are expressed in astrocytes (6, 7). A variety of studies also indicate that the activation of CB_1_R or CB_2_R produced effects of anti-inflammation, antinociception, and neuroprotection (8), and activation of MAPK signaling (9). In addition, activation of CB_1_R or CB_2_R can inhibit the activation of MAPK cascade induced by stress, and the discrepancy remains to be further studied. Our recent study indicates that astrocytic MAPK subunits, extracellular signal-regulated protein kinase ½ (ERK1/2) and p38, are involved in the modulation of GS by CB_1_R and CB_2_R (10). Other studies indicate that c-Jun N-terminal kinase (JNK) participates in the CB_1_R-mediated inflammation signaling (11) and CB_2_R-mediated suppression of leukocyte migration under inflammation (12). However, there is no study about whether JNK participates in the modulation of astrocytic GS by eCBs. Taking into consideration the importance of cannabinoids (CBs) and astrocytic GS in chronic inflammatory pain, it is necessary to eliminate the role of JNK in astrocytic GS in CB-mediated chronic inflammation, which may be helpful for therapeutic strategy.

## Materials and Methods

This study was approved by the Ethics Committees of Animal Usage of Lanzhou University, Southern Medical University, and Shenzhen University.

### Primary Cultures of Astrocyte

The primary culture of astrocytes was performed as described previously (10). In brief, the newborn Sprague-Dawley **(**SD) rats (postnatal 1–3 days) from the experimental animal center of the Gansu University of Chinese Medicine were decapitated and the cerebral hemispheres were aseptically harvested into Hank's balanced salt solution (HBSS). After the removal of meninges, the cerebral cortices were trimmed into small pieces, followed by digestion with 0.25% trypsin-ethylenediaminetetraacetic acid (EDTA) (Gibco Life Technology, CA, USA), mechanical dissociation by gentle pipetting with Pasteur pipette, and then centrifugation at 400 g for 5 min. The cells were resuspended in a culture medium supplemented with 90% Dulbecco's Modified Eagle Medium/Nutrient Mixture F-12 (DMEM/F12) (Gibco Life Technology, CA, USA) and 10% fetal bovine serum (FBS; PAN-Biotech, Germany) and plated at a density of 3–5 × 10^5^ cells/cm^2^ in 25 cm^2^ flasks. Cells in flasks were cultured at 37°C in a carbon dioxide (CO_2_) incubator for 5–7 days to reach the first confluence. To achieve high pure astrocytes (>95%), the confluent cells in flasks were shaken at 200 rpm overnight to diminish contamination from microglia. Afterward, the astrocytes were evenly passaged into 35 mm dishes and treated with 1 μg/ml lipopolysaccharide (LPS), which is one commonly used chemical to induce inflammation and can activate astrocytes via the JNK signaling pathway (13), JNK phosphorylation inhibitor SP600125, or with 0.01 μM 2-AG.

### Protein Isolation and Western Blotting

According to the previous report, the Western blotting was carried out with the manual (10). In brief, astrocytes in 35 mm dishes were lysed in 100 μl radioimmunoprecipitation assay (RIPA) lysis buffer containing 1% phenylmethanesulfonyl fluoride (PMSF) after different treatments. Lysates were centrifuged at 12,000 rpm for 10 min to remove cell debris, and the pellet was diluted with 30 μl sample buffer. The total protein in lysates was measured for concentration by bicinchoninic acid (BCA) and loaded onto 10% sodium dodecyl sulfate (SDS)-polyacrylamide gels at 5–20 μg/lane and then separated by electrophoresis and transferred to polyvinylidene difluoride (PVDF) membranes. Following the non-specific binding sites blockade with 5% non-fat milk in Tris-buffered saline with Tween-20 (TBST) for 2 h at room temperature (RT), the PVDF membranes were incubated overnight at 4°C with primary antibodies according to the manual of the manufacturer [at a dilution of 1:1,000 for JNK and phospo-JNK (p-JNK) antibodies, #9252 and #9255, Cell Signaling Technology, MA, USA; or 1:10,000 for GS antibody; #ab176562, Abcam, St. Louis, MO, USA] and then washed extensively with TBST three times, 10 min for each time, and incubated with corresponding secondary antibodies (1:10,000; Cell Signaling Technology, MA, USA) at RT for 2 h. The membranes were then washed three times with TBST at 10-min intervals, and the immunolabeled protein bands on membranes were detected by using an enhanced chemiluminescence kit.

### Immunocytochemistry

After different treatments, the astrocytes cultured on coverslips were fixed with 4% paraformaldehyde for 30 min and rinsed with phosphate-buffered saline (PBS). The fixed cells were then permeabilized with 0.4% Triton X-100 for 20 min, rinsed again with PBS, incubated with 3% normal goat serum (NGS) for 30 min, and then incubated with different primary antibodies (GS, 1:5,000; JNK and p-JNK, 1:500) overnight at 4°C, respectively. After 24 h, the coverslips were rinsed with PBS and incubated with corresponding secondary antibodies conjugated with Alexa Fluor® 488 (green staining) or 594 (red staining) (Invitrogen, UK) for 2 h at RT. Then, the coverslips were mounted onto the slide with a mounting medium with 4′,6-diamidino-2-phenylindole (DAPI) for the observation of nuclei and sealed with nail gel. The cells were visualized by immunofluorescence microscope (Olympus, Japan).

### Statistical Analysis

All experiments were performed in triplicate and repeated at least three times. STATA software version 14.2 (Stata Corp, College Station, TX, USA) was used for statistical analysis, and the data were expressed as the mean ± SEM. One-way ANOVA followed by the Newman-Keuls test was used to assess the significant differences, and *p* < 0.05 was considered as significantly different.

## Results

### Lipopolysaccharide (LPS) Activated Expression of GS and Phosphorylation of JNK and Translocation in Primary Astrocytes of Rats

**Figure 1:**
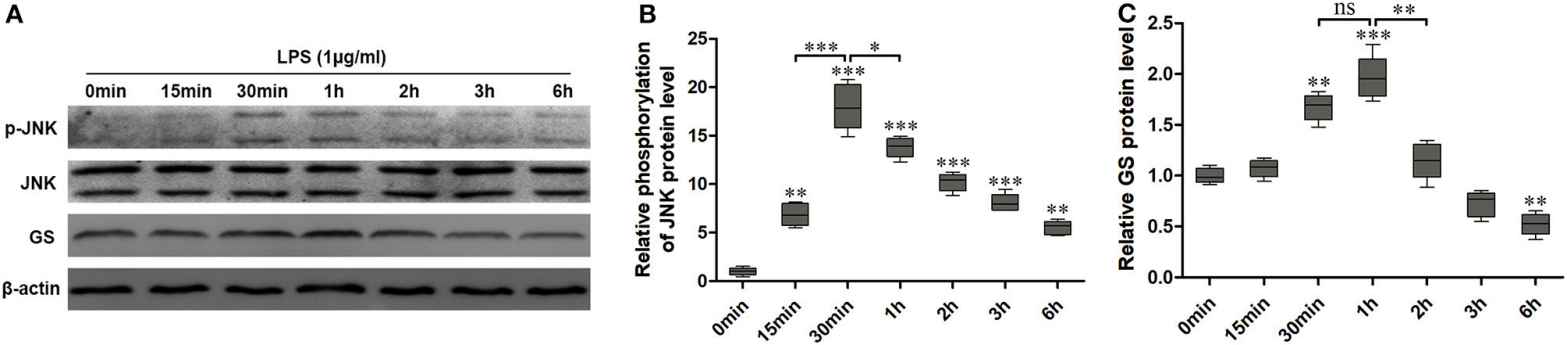
Exposure to lipopolysaccharide (LPS) induced changes in the expression of glutamine synthetase (GS) and activation of c-Jun N-terminal kinase (JNK). Astrocytes were treated with 1 μg/ml LPS for different times, and protein levels of GS **(A,B)** and phospo-JNK (p-JNK) **(A,C)** were analyzed using Western blotting. Error bars were ± SEM. *n* = 3. *p < 0.05, **p < 0.01, and ***p < 0.001 vs. control. ns, not significant.

**Figure 2:**
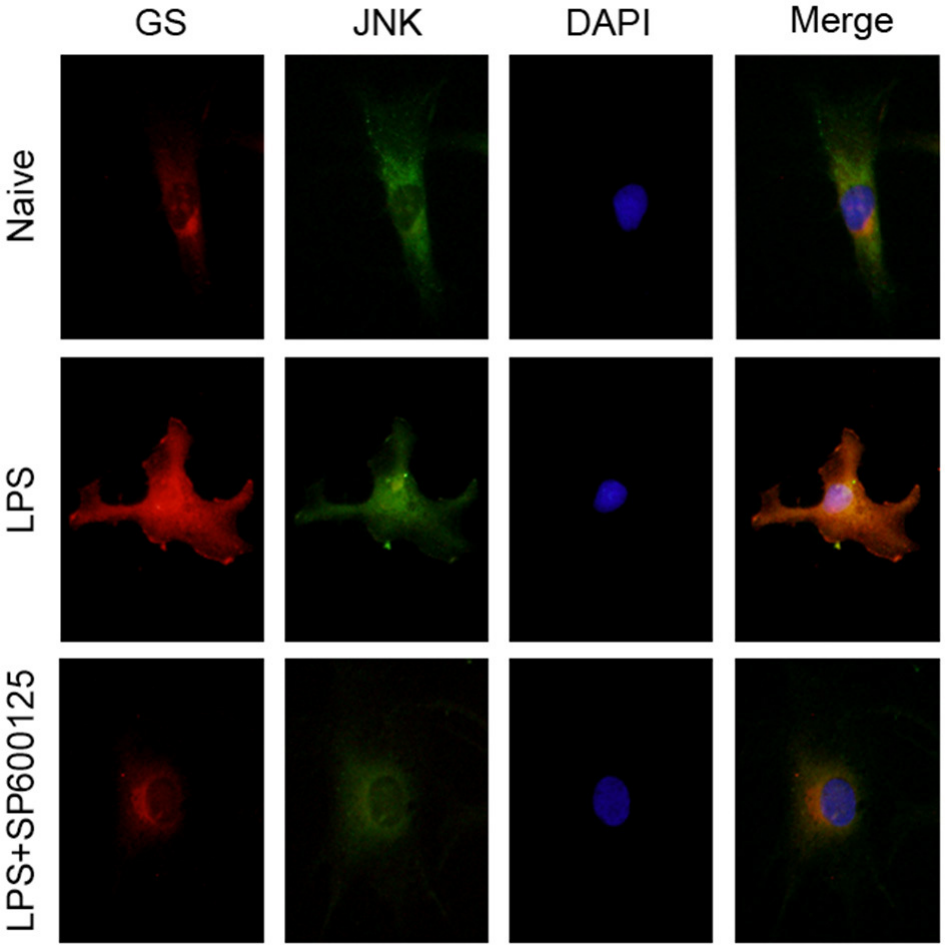
Inhibition of phosphorylation of JNK prevented the translocation of JNK and upregulation of GS induced by exposure to LPS. Astrocytes were pretreated with SP600125 for 1 h and exposed to 1 μg/ml LPS for 1 h. Immunocytochemistry assay was used to analyze the translocation of JNK and changes in the expression of GS. Scar bar = 10 μm.

Similar to our previous study (10), 1 μg/ml LPS was used to activate the astrocytes. To investigate the effects of LPS on the JNK pathway and expression of GS in astrocytes, the expressions of p-JNK, JNK, and GS in primary astrocyte culture were evaluated using Western blotting after treatment with DMEM/F12 containing 1 μg/ml LPS at 0 min, 15 min, 30 min, 1 h, 2 h, 3 h, and 6 h. The data showed that the exposure to LPS induced time-dependent biphasic changes in the expression of GS in astrocytes, i.e., in contrast to baseline (0 min), expression of GS began to increase at 30 min (1.62 ± 0.08, *p* < 0.01), peaked at 1 h (1.86 ± 0.08, *p* < 0.001), declined to control level at 2–3 h, and then decreased at 6 h (0.57 ± 0.05, *p* < 0.01; Figures 1A,B). Regarding the JNK pathway, LPS significantly increased the phosphorylation of JNK in a time-dependent manner while without effect on the total JNK. The protein level of p-JNK increased at 15 min (6.96 ± 0.63, *p* < 0.01), reached a maximal level at 30 min (18.12 ± 0.90, *p* < 0.001), and then gradually declined but was still higher at 6 h (5.60 ± 0.49, *p* < 0.01) than control (Figures 1A,C). In addition, previous studies indicated that the JNK pathway exerted its role through translocation from the cytoplasm to nucleus (14). As expected, 1 μg/ml LPS for 1-h exposure induced the translocation of JNK from cytoplasm to nucleus, which was prevented by JNK phosphorylation inhibitor SP600125 (Figure 2). In addition, SP600125 also prevented the expression of GS by LPS.

### 2-Arachidonoylglycerol (2-AG) Reversed Changes in LPS-Induced GS Independently on the p-JNK Pathway

**Figure 3:**
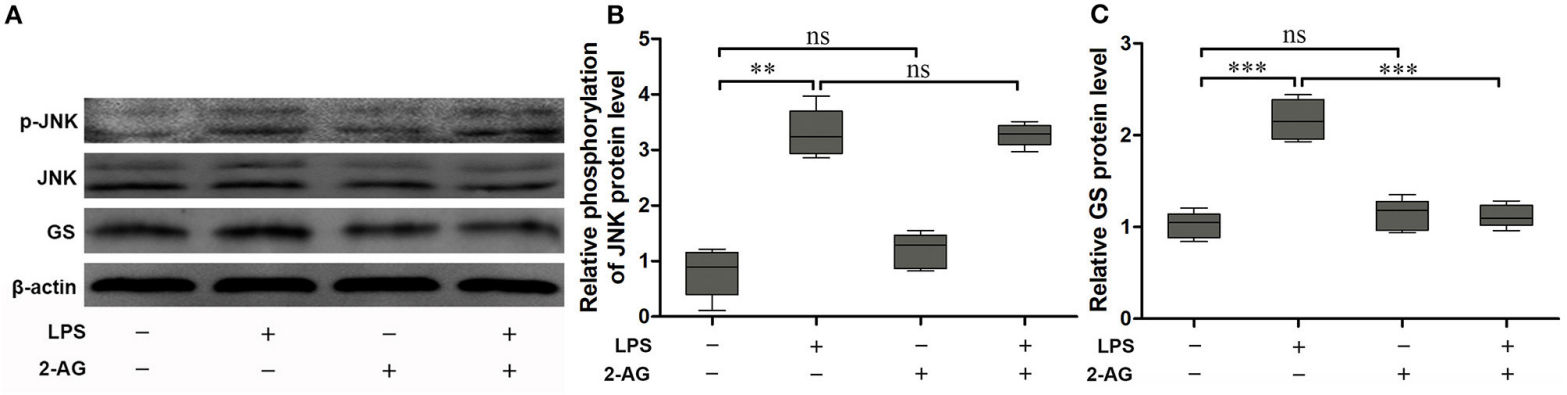
2-Arachidonoylglycerol (2-AG) suppressed the upregulation of expression of GS but had no effect on phosphorylation of JNK induced by exposure to LPS in astrocytes. Astrocytes were pretreated with 0.01 μM 2-AG for 2 h and exposed to 1 μg/ml LPS for 1 h. The protein levels of GS **(A,B)** and p-JNK **(A,C)** were measured using Western blotting. Error bars were ± SEM. *n* = 3. **p < 0.01, and ***p < 0.001. ns, not significant.

To explore the effects of 2-AG on the activation of astrocytes induced by exposure to LPS, the astrocytes were exposed to 1 μg/ml LPS for 1 h and were chosen on the basis of the acquired data from Figure 1. The cells were pretreated with 1 μM 2-AG for 1 h and/or 1 μg/ml LPS for 1 h. Compared with control (0 μg/ml LPS), exposure of astrocytes to 1 μg/ml LPS for 1 h significantly elevated the expressions of p-JNK (2.18 ± 0.18, *p* < 0.01) and GS (3.41 ± 0.29, *p* < 0.01) and treatment with 2-AG could significantly reverse the changes in the expression of GS induced by exposure to LPS (1.13 ± 0.09, *p* < 0.01) when compared with LPS group (Figures 3A,B) but without significant effect on the expression of p-JNK (Figures 3A,C).

### Dephosphorylation of JNK Increased the Expression Level of GS

**Figure 4:**
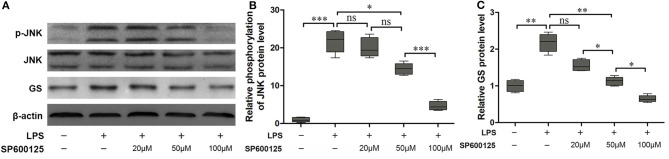
Inhibition of phosphorylation of JNK prevented the effect of LPS-induced upregulation of expression of GS. Astrocytes were pretreated with SP600125 for 1 h and exposed to 1 μg/ml LPS for 1 h. The protein levels of GS **(A,B)** and p-JNK **(A,C)** were measured using Western blotting. Error bars were ± SEM. *n* = 3. *p < 0.05, **p < 0.01, and ***p < 0.001. ns, not significant.

According to the above results, phosphorylation of JNK is not the pathway of 2-AG modulating the expression of GS in astrocytes. To further address the question of whether the JNK pathway was involved in the process of regulating the expression of GS, a specific inhibitor for the JNK signaling pathway, SP600125, was used to investigate the relationship between the JNK pathway and the expression of GS in astrocytes. SP600125 at the concentration of 50 μM and 100 μM could significantly inhibit LPS-induced activation of JNK in a dose-dependent manner (16.71 ± 0.75, *p* < 0.05; 5.03 ± 0.33, *p* < 0.001) when compared with LPS alone (22.41 ± 1.18) (Figures 4A,C). Meanwhile, SP600125 at the concentration of 50 and 100 μM could also significantly suppress the LPS-induced upregulation of expression of GS in a dose-dependent manner (1.15 ± 0.84, *p* < 0.01; 0.79 ± 0.06, *p* < 0.01) when compared with LPS alone (2.14 ± 0.15) (Figures 4A,B). In addition, SP600125 also inhibited the translocation of p-JNK (Figure 2). Briefly, these data suggested that LPS activated JNK resulting in the upregulation of expression of GS. In the other words, GS was the downstream target of JNK signaling in astrocytes.

## Discussion

Previous studies indicate that GS is involved in suppressing the development of glutamate/ammonia neurotoxicity and a variety of neurological diseases, such as neuropathic pain and inflammatory pain (1). Intriguingly, both the increase and the decrease of GS are reported in the same diseases, such as hepatic encephalopathy, traumatic brain injury, and epilepsy, but contrarily, controlling the expression of GS can diminish these diseases. Consistent with our previous study (10), this study finds that exposure to LPS resulted in the expression of GS in a biphasic form in astrocytes, i.e., the expression of GS is increased with short-term exposure to LPS and decreased with long-term exposure to LPS.

The MAPK family of kinases including p38 and ERK participate in pain and neurodegenerative diseases and exist in activated astrocytes induced by pathological stimulation (6). Our previous study indicates that exposure to LPS could activate p38 and ERK1/2 in astrocytes with different patterns (10). In this study, we find that inhibition of JNK blocks the increase in GS by LPS, of which the mechanism may be through suppression of glucocorticoid receptor transcriptional activity (15). This study further indicates that exposure to LPS produces a uniphasic activation of JNK in astrocytes, which enriches the involvement of MAPK signaling in LPS-induced changes in GS in astrocytes. However, the mechanism remains to be further studied.

2-Arachidonoylglycerol (2-AG) is an eCB that binds to CB_1_R and CB_2_R expressed in astrocytes. The previous study found that astrocytic p38 can be activated by 2-AG, while blocking CB_1_R can produce an inhibitory effect on the modulation of 2-AG on p38 (9), implying that 2-AG participates in the modulation of MAPK signaling in astrocytes. Our previous study suggests that, under the condition of short-term exposure to LPS, activation of p38 could increase the expression of GS, and 2-AG could suppress the increased expression of GS by inhibiting the phosphorylation level of p38. While under the condition of long-term exposure to LPS, activation of ERK1/2 results in a decrease of expression of GS and 2-AG reverses the decrease of expression of GS through reducing the activation of ERK1/2 (10). It should be noted, in our previous study, that although ERK1/2 and p38 are activated by short-term exposure to LPS and long-term exposure to LPS, respectively, the activation is relatively weaker compared to long-term and short-term exposure to LPS, respectively. This study indicates that JNK is activated during the short-term exposure to LPS, while 2-AG has no effect on the phosphorylation of JNK. However, other studies indicate that JNKs are involved in the CB_1_R-mediated inflammation signaling (11) and CB_2_R-mediated suppression of leukocyte migration under inflammation (12). These results imply that activation of JNK may be the upstream pathway of the astrocytic extracellular matrix (ECM) system in modulating inflammation, which remains to be further investigated.

In conclusion, this study indicated that exposure to LPS for the short term and long term can produce different changes in the activities of GS in astrocytes with activation of JNK. ECB 2-AG modulates the expression of GS induced by exposure to LPS, which is not dependent on the activation of JNK. The mechanism of JNK in modulating the astrocytic expression of GS remains to be further studied.

## Data Availability Statement

The original contributions presented in the study are included in the article/supplementary material, further inquiries can be directed to the corresponding author/s.

## Ethics Statement

The animal study was reviewed and approved by the Animal Ethic Committee of Lanzhou University.

## Consent for Publication

The authors all consented to publication.

## Author Contributions

SW performed the experiments and analyzed the data. HZ analyzed the data. JW designed the experiments and wrote the manuscript. All authors contributed to the article and approved the submitted version.

## Conflict of Interest

The authors declare that the research was conducted in the absence of any commercial or financial relationships that could be construed as a potential conflict of interest.

## Abbreviations

2-AG: 2-arachidonylglycerol
CBR: cannabinoid receptor
ERK1/2: extracellular signal-regulated protein kinase 1/2
GS: glutamine synthetase
LPS: lipopolysaccharide
MAPK: mitogen-activated protein kinase.
